# Exploratory Serum-Based Surface-Enhanced Raman Spectroscopy Analysis in Crohn’s Disease: A Pilot Cross-Sectional Study

**DOI:** 10.3390/ijms27125180

**Published:** 2026-06-08

**Authors:** Dan Vălean, Roxana Zaharie, Valentin Toma, Anca Onaciu, Rareș-Mario Borșa, Rareș-Ionuț Știufiuc, Alin Fetti, Beata Dohi, Călin Popa, Emil Moiș, Andra Ciocan, Nadim Al-Hajjar, Florin Zaharie

**Affiliations:** 1 Department of Surgery, “Iuliu Hațieganu” University of Medicine and Pharmacy, 400012 Cluj-Napoca, Romania; valean.d92@gmail.com (D.V.); dr.alinfetti@yahoo.com (A.F.); dohibeata@yahoo.com (B.D.); calinp2003@yahoo.com (C.P.); drmoisemil@gmail.com (E.M.); andra.ciocan10@gmail.com (A.C.); na_hajjar@yahoo.com (N.A.-H.); florinzaharie@yahoo.com (F.Z.); 2 Regional Institute of Gastroenterology and Hepatology “Octavian Fodor”, 400162 Cluj-Napoca, Romania; 3 MEDFUTURE—Institute for Biomedical Research, Department of NanoSciences, “Iuliu Hațieganu” University of Medicine and Pharmacy, 400349 Cluj-Napoca, Romania; valentin.toma@umfcluj.ro (V.T.); anca.onaciu@umfcluj.ro (A.O.); rares.mari.borsa@elearn.umfcluj.ro (R.-M.B.); rares.stiufiuc@umfcluj.ro (R.-I.Ș.); 4 Pharmaceutical Physics—Biophysics, Department Pharmacy 1, Faculty of Pharmacy, “Iuliu Hațieganu” University of Medicine and Pharmacy, 400349 Cluj-Napoca, Romania; 5 Maxillofacial Surgery and Implantology, Department 1—Maxillo-Facial Surgery and Radiology, Faculty of Dental Medicine, “Iuliu Hațieganu” University of Medicine and Pharmacy, 400029 Cluj-Napoca, Romania; 6 Dental Propedeutics and Esthetics, Department 4—Prosthetic Dentistry and Dental Materials, Faculty of Dental Medicine, “Iuliu Hatieganu” University of Medicine and Pharmacy, 400029 Cluj-Napoca, Romania

**Keywords:** RAMAN, SERS, blood serum, molecular discrimination, Crohn’s disease, cohort study

## Abstract

Crohn’s disease (CD) is a chronic inflammatory bowel disease requiring accurate and timely diagnosis. Current diagnostic tools may be invasive, costly, or insufficiently specific. Surface-enhanced Raman spectroscopy may enable rapid, minimally invasive detection of disease-associated biochemical alterations in serum. This cross-sectional pilot study included age- and sex-matched patients with Crohn’s disease and healthy controls. Serum samples were analyzed using surface-enhanced Raman spectroscopy. Spectral data were preprocessed and analyzed using principal component analysis-linear discriminant analysis and partial least squares-discriminant analysis. Classification performance was evaluated using leave-one-out cross-validation. Variable importance in projection scores was used to identify discriminatory vibrational bands. Fifty-four participants fulfilled the clinical inclusion criteria, while 51 participant-level spectra were retained for final classification analysis. PCA-LDA differentiated CD from healthy controls with a sensitivity of 85.19%, specificity of 91.67%, accuracy of 88.24%, and AUC of 0.881. PLS-DA showed slightly higher performance, with a sensitivity of 88.89%, specificity of 95.83%, accuracy of 92.16%, and AUC of 0.937. Relevant discriminatory bands were observed at 498, 639, 728, 813, 1136, 1205, 1443, 1579, and 1657 cm^−1^, suggesting alterations in purine metabolism, protein structure, lipid composition, and nucleic acid-associated signals. Serum-based SERS combined with multivariate analysis showed promising ability to distinguish patients with CD from healthy controls in this pilot cohort. Larger multicenter studies are required to validate these findings and assess clinical applicability.

## 1. Introduction

Crohn’s disease is a relapsing inflammatory bowel disease that can affect any segment of the gastrointestinal tract and is associated with a progressive, heterogeneous clinical course presenting with a high degree of morbidity [1]. Diagnosing and staging CD can be itself a challenge, due to the high prevalence of autoimmune diseases that can cause gastrointestinal lesions which can mimic IBD, in addition to the high degree of variability in the clinical, imaging as well as endoscopic findings in these patients. This can sometimes lead to increased costs in diagnosing in addition to adequately treating the individuals [2]. Furthermore, the current biomarkers and imaging tests used in diagnosing IBD (Inflammatory Bowel Diseases) have a certain degree of limitations, as they have limited specificity, sometimes not being able to distinguish IBD from various bacterial inflammations of the digestive tract as well as other auto-immune diseases [3].

Given the increasing prevalence of Crohn’s disease among younger individuals and its changing global epidemiology, particularly in newly industrialized regions, early diagnosis remains essential to ensure timely treatment and reduce long-term complications [4,5]. In spite of the significant improvements performed in diagnosing, treating and staging inflammatory bowel diseases, there is still a need in developing specific, adequate as well as cost-effective diagnostic tests [6]. Recent developments in multiomics have paved the way of new methods of early diagnosis and a better understanding in the etiopathogeny of CD as well as individualizing the potential evolution of the disease, however these methods of early diagnosis need to be standardized in order to achieve feasibility [7].

Raman spectroscopy is an optical vibrational spectroscopy technique that enables molecular characterization based on the inelastic scattering of monochromatic laser light by biological samples. The resulting spectra provide highly specific biochemical fingerprints that reflect the molecular composition and structural changes of tissues and biofluids. Owing to its non-destructive nature, minimal sample preparation requirements, and ability to detect subtle biochemical alterations associated with disease states, Raman spectroscopy has found broad applications in medicine, biology, and physics. However, the clinical utility of conventional Raman spectroscopy is limited by the intrinsically weak intensity of Raman scattering, particularly when analyzing complex biological matrices such as serum or plasma.

Surface-enhanced Raman spectroscopy (SERS) is an advanced Raman-based technique that addresses this limitation by amplifying Raman signals through the interaction of analytes with nanostructured metallic surfaces, most commonly silver or gold nanoparticles. This enhancement enables the ultrasensitive detection of low-abundance biomolecules and has expanded the potential clinical applications of Raman-based diagnostics [8].

Despite its considerable diagnostic potential, SERS still faces several limitations that restrict its routine clinical implementation. Signal enhancement depends strongly on the physicochemical properties of metallic nanostructures; therefore, variations in nanoparticle size, morphology, surface chemistry, and hotspot distribution may affect spectral reproducibility and contribute to inter-laboratory variability. Furthermore, complex biological matrices such as serum, plasma, or fecal samples may generate matrix effects and nonspecific molecular adsorption, potentially obscuring disease-associated spectral features and complicating interpretation [9]. Although SERS substantially improves analytical sensitivity compared with conventional Raman spectroscopy, quantitative analysis remains challenging, particularly for longitudinal disease monitoring. In addition, spectral acquisition is highly sensitive to experimental conditions, including laser wavelength, laser power, acquisition time, substrate preparation, and sample handling procedures, which continues to limit methodological standardization across clinical settings [10].

Nevertheless, these characteristics make SERS a promising diagnostic tool for inflammatory bowel diseases, including Crohn’s disease. By enabling the detection of disease-specific molecular signatures in biological specimens, SERS may facilitate early diagnosis, disease stratification, and treatment monitoring. Its potential utility in inflammatory bowel disease, particularly ulcerative colitis, has already been demonstrated in studies involving colon tissue specimens, while similar approaches have shown promising results in other diseases, including breast and oral cancers using blood-derived samples [11,12,13].

This study aimed to evaluate whether serum SERS combined with multivariate analysis can discriminate patients with CD from healthy controls. We hypothesize that SERS biofluid analysis can distinguish CD subjects from non-CD subjects with high accuracy.

## 2. Results

### 2.1. Subject Data and Disease Classification

All 54 patients that were included in the study fulfilled the matching criteria. In both groups, 74.1% of the patients were male (*n* = 20). Patients were aged 21–59, with a mean age of 31.47 (±5.14). All included patients were known for ileo-colic CD. Twelve patients were in remission (CDAI < 150) while 15 patients were with low to moderate activity (CDAI < 220). Further data are highlighted in Table 1. Statistically significant differences were highlighted between the Mean leukocytes and mean CRP levels.

### 2.2. Univariate and Multivariate Analysis of SERS

**Figure 1:**
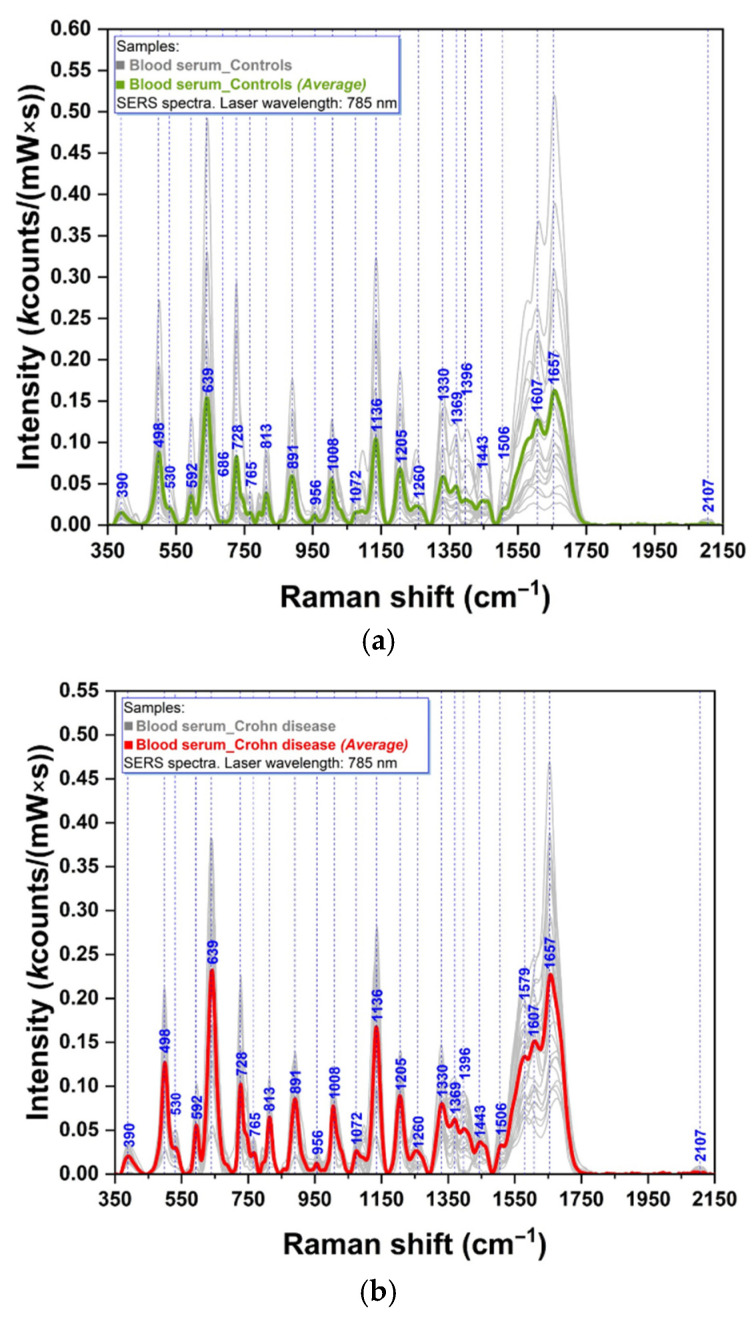
(**a**) Mean compared spectra for controls. (**b**) Mean compared spectra for CD patients.

Mean compared spectra alongside the standard deviation, for both groups are shown in Figure 1a,b. When performing multivariate analysis, multiple technical replicate spectra were acquired from each serum sample. After preprocessing, replicate spectra were averaged to generate a single representative spectrum for each participant.

**Figure 2:**
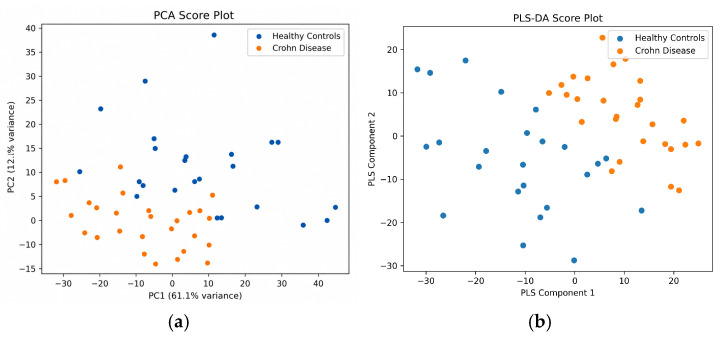
(**a**) PCA score plot. (**b**) PLS-DA score plot.

PCA was used to reduce spectral dimensionality before LDA classification. The number of principal components remained was selected to explain 99% of variance within the training set. PC1 accounted for 61% of total variance and was mainly associated with spectral contributions at 1657 cm^−1^ and 639 cm^−1^. The PCA score plot showed partial separation between CD and healthy control (Figure 2a,b).

**Figure 3:**
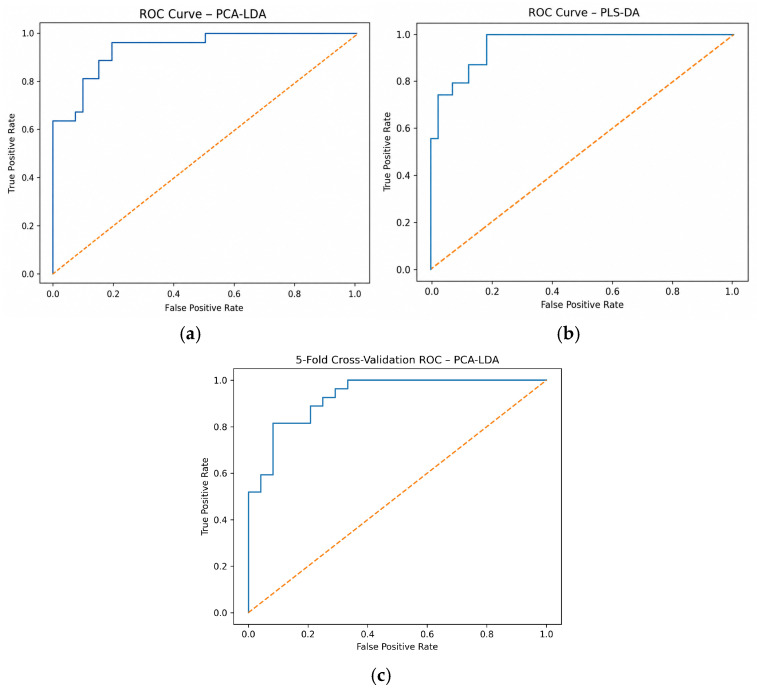
(**a**) ROC curve PCA-LDA. (**b**) ROC curve PLS-DA. (**c**) 5-fold Cross-Validation ROC—PCA-LDA.

Although 54 participants were included, 51 participant-level averaged spectra were retained for classification analysis (27 CD, 24 healthy patients). Classification performance was assessed using PCA-LDA and PLS-DA models, separation was performed using a Leave-One-Out Cross-Validation (LOOCV) protocol, ensuring that each spectrum served as a test set exactly once. As a result of this procedure, samples from CD patients could be differentiated from healthy samples with a sensitivity of 85.19%, specificity of 91.67%, and an accuracy of 88.24%. The predictive value of the technique was performed using a ROC analysis, calculating the area under the curve (AUC), in our study, the AUC was 0.881 (Figure 3a).

Similarly, the PLS-DA analysis, yielded a sensitivity rate of 88.89%, specificity rate of 95.83% with an accuracy of 92.16%, with an AUC of 0.937 (Figure 3b). Therefore, in comparison, the PLS-DA model, achieved slightly superior accuracy, focusing on the disease specific covariance. Results are summarized in Table 2. To further evaluate model robustness, an additional stratified 5-fold cross-validation analysis and permutation testing were performed. The PCA-LDA model maintained high discriminatory performance during repeated internal validation (AUC = 0.931), while permutation testing demonstrated that the observed classification performance was significantly greater than expected by chance (*p* < 0.001, Figure 3c).

**Figure 4:**
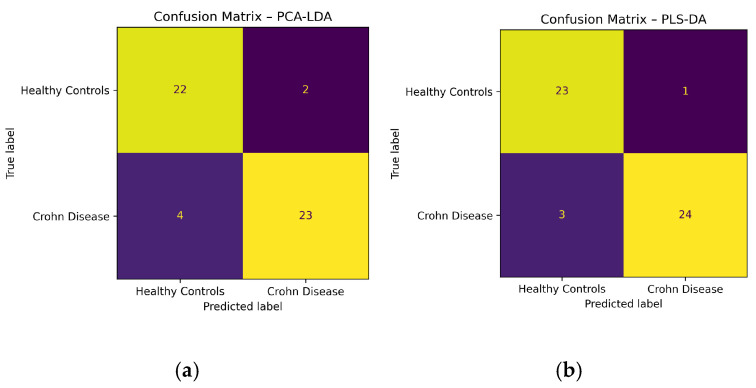
(**a**) Confusion Matrix for PCA-LDA. (**b**) Confusion Matrix for PLS-DA.

To further evaluate classification performance at the individual sample level, confusion matrix analysis was performed for both PCA-LDA and PLS-DA models following LOOCV classification. The PCA-LDA model correctly classified 23 of 27 Crohn’s disease samples and 22 of 24 healthy control samples, corresponding to a sensitivity of 85.19% and specificity of 91.67%. Similarly, the PLS-DA model correctly identified 24 of 27 Crohn’s disease samples and 23 of 24 healthy controls, achieving a sensitivity of 88.89% and specificity of 95.83%. Overall, the confusion matrices demonstrated a low misclassification rate and supported the strong discriminatory performance observed in ROC analysis, particularly for the PLS-DA model (Figure 4a,b).

### 2.3. Identification of Vibrational Bands

**Figure 5:**
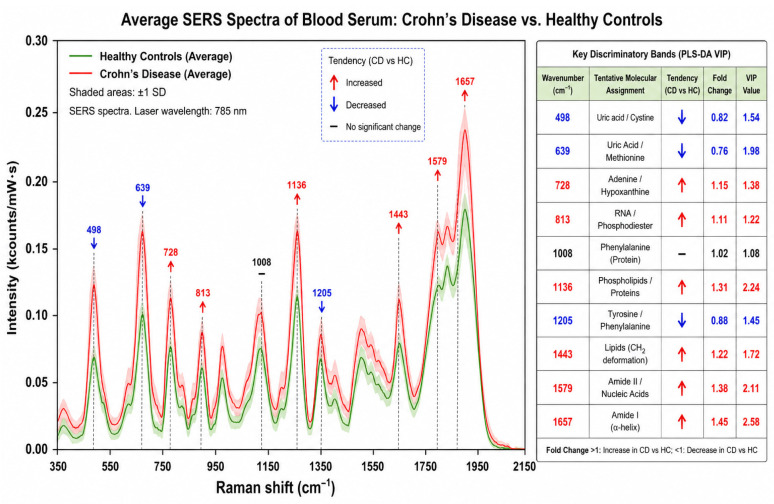
SERS Spectra and VIP between CD and Healthy Controls.

Variable Importance in Projection (VIP) scores were calculated to determine the contribution of each wavenumber to the classification model. Wavenumbers with VIP > 1 are considered highly significant (Figure 5).

Statistical significance was further validated via *t*-tests with Benjamini-Hochberg (BH) correction at a 5%False Discovery Rate (FDR—*p* < 0.0336). The corresponding values of maximum importance are highlighted in Table 3. Based on the available data in the literature, tentative identification of the numbers is also present [8,14,15,16,17,18,19,20,21,22,23,24,25,26,27,28].

## 3. Discussion

This study emphasizes that SERS can detect molecular alterations in patients with CD with low to moderate activity compared to healthy patients with a high degree of sensitivity, as reported in literature [29,30]. SERS may enhance the detection of molecular patterns that are difficult to identify using conventional Raman spectroscopy. One of the main advantages is that it does not require extensive sample preparation, thus obtaining faster results as well as lowering the preparation costs. Compared with histological assessment, which may require several days, serum-based SERS could potentially provide faster adjunctive molecular information. However, interpretation of SERS spectra remains challenging because not all metabolites and vibrational bands have been fully characterized, particularly in complex biological samples. Despite these limitations, subtle spectral differences may still contain valuable diagnostic information when analyzed using appropriate multivariate statistical methods. Such approaches facilitate the identification of clinically relevant patterns and group discrimination, even when the assignment of individual spectral peaks remains incomplete. Nevertheless, the need for advanced spectral interpretation and ongoing characterization of vibrational bands continues to represent a limitation of the technique. The self-assembled AgNP SERS platform employed in this study has demonstrated analytical robustness and applicability in several previous biomedical investigations conducted by our research team [31,32].

In our study, the statistically significant decreased levels at 438 and 639 cm^−1^, may indicate a depletion of the systemic antioxidant capacity. These peaks are usually attributed to uric acid, which is a terminal product of the purine catabolism, and a major antioxidant [33,34]. Thus, the high VIP values (1.54 and 1.98 respectively) highlight the antioxidant imbalance which may be a primary driver in discriminating CD from healthy patients.

In addition, the consistent significant increase in the hypoxanthine signal (728 cm^−1^) is one of the relevant indicators of the metabolic imbalance occuring in the inflamed bowel wall, thus highlighting increased purine catabolism associated with inflammatory and hypoxic tissue stress.

The most significant diagnostic features identified in the current dataset were the Amide I and II bands (1657 and 1579 cm^−1^), which exhibited the highest VIP values and among the largest fold changes (1.38 and 1.45, respectively). The increased intensity of these protein-associated bands may reflect protein remodeling related to systemic inflammation and catabolic metabolic alterations in Crohn’s disease. These findings are consistent with the release of acute-phase proteins and unfolded proteins into the circulation, potentially driven by elevated matrix metalloproteinase activity during intestinal remodeling. Similarly, the increased phospholipid-associated signal at 1136 cm^−1^ may indicate enhanced cell membrane turnover and mobilization of lipid mediators involved in inflammatory processes. Elevated nucleic acid-associated bands may reflect increased cellular turnover and inflammatory tissue injury. In contrast, the reduced intensity observed at 1205 cm^−1^, attributed to the aromatic amino acids tyrosine and phenylalanine, may be consistent with systemic amino acid mobilization supporting immune cell proliferation and inflammatory mediator synthesis [35,36,37]. Although the phenylalanine-associated band at 1008 cm^−1^ did not remain statistically significant after correction for multiple comparisons, it was retained because of its relative spectral stability and its established role as an internal spectral reference in biological Raman and SERS studies [38].

The high predictive accuracy observed in this pilot cohort supports further investigation of serum SERS as a potential adjunctive screening tool. Similar diagnostic approaches have been explored using urine and plasma samples, highlighting the broader applicability of SERS for disease detection and treatment monitoring [33]. Furthermore, compatibility with portable spectrometers may facilitate implementation in clinical settings, potentially enabling rapid diagnosis and risk stratification at the point of care [34,39,40,41].

To our knowledge, this is the first study using blood serum to discriminate Crohn’s disease from healthy subjects.

However, despite the promising results, some challenges remain, and the limitations of the present study should be acknowledged. First, this was a single-center pilot study with a relatively small sample size, which may limit the robustness and generalizability of the classification models. Although leave-one-out cross-validation was employed to maximize data utilization, the absence of an independent external validation cohort increases the risk of optimistic performance estimates and potential overfitting. Second, only healthy controls were included, without additional disease-control groups such as ulcerative colitis, infectious colitis, irritable bowel syndrome, celiac disease, or other inflammatory and autoimmune conditions. Therefore, the observed spectral differences should not be interpreted as Crohn’s disease-specific molecular signatures, as part of the discrimination may reflect broader systemic inflammatory or metabolic alterations. In addition, the biochemical interpretation of several Raman bands remains tentative because no independent metabolomic or biochemical validation methods, such as LC-MS, ELISA, or oxidative stress profiling, were performed. Another important limitation relates to the intrinsic variability of nanoparticle-based SERS substrates, including potential differences in hotspot distribution and signal enhancement reproducibility, despite the use of standardized preparation and acquisition protocols. Finally, correlations between spectral features and inflammatory indices such as CRP, leukocyte count, fecal calprotectin, CDAI, or SES-CD were not evaluated in the present cohort and should be explored in future longitudinal multicenter studies. The classification performance in addition to peak spectra levels can be influenced by fluctuations in biofluid composition, together with deviations from the protocol may influence the results. This justifies the excluded patients due to deviations from the standard protocol (e.g., blood harvesting, different nurse), and the matching criteria, to limit the risk of bias, thus underlying one of the study’s strengths. Another limitation of our study remains in the sample size. These results need to be validated either in a larger sample size, or a multicentric study. In addition, although LOOCV is appropriate for small datasets, it may yield optimistic estimates of model performance. Independent validation in external cohorts is required.

In addition, this is a single center study, which encompasses a specific geographic region and a specific disease phenotype (Romania, Eastern Europe). Further studies on larger sample sizes with a more complex differentiation and a more thorough serological profile are required in order to adequately discriminate between inflammatory bowel diseases, however this study has achieved a diagnostic serological profile of CD. The results described here not only can prove useful in a faster, cheaper method of diagnosing CD, but may allow a more thorough differentiation of other inflammatory bowel diseases as well as undifferentiated inflammatory disease, or undetermined colitis from other auto-immune pathologies. Because only healthy controls were included, the current findings cannot yet establish disease specificity relative to ulcerative colitis, infectious colitis, irritable bowel syndrome, or other inflammatory conditions. Lastly, the model was trained exclusively on patients with mild-to-moderate ileocolonic Crohn’s disease, which may limit generalizability to severe or isolated small bowel disease. Further studies are required in this regard. The current findings should not be interpreted as evidence of Crohn’s disease-specific molecular signatures, since additional inflammatory and gastrointestinal disease controls were not included.

## 4. Materials and Methods

### 4.1. Subject Data

This cross-sectional pilot study was approved by the local institutional ethical review board (Comisia de Etică a Universității de Medicină și Farmacie “Iuliu Hațieganu”—approval no. 207/2024). The study protocol conforms to the ethical guidelines of the 1975 Declaration of Helsinki as reflected in a priori approval by the institution’s human research committee.

70 patients were enrolled in the study, divided into two groups: Group A (Healthy Group, *n* = 35), consisting of healthy patients, and Group B (Crohn Group, *n* = 35) consisting of the patients diagnosed with CD. Patients were recruited from the Regional Institute of Gastroenterology and Hepatology “Octavian Fodor” Cluj-Napoca. Inclusion criteria were established for both groups and individuals. The primary inclusion criteria consisted of patients aged over 18, with no other auto-immune conditions or previously known malignancies. Patients with CD were evaluated using the Crohn Disease Activity Index (CDAI) which evaluates several criteria, totaling a maximum of 600 points [42]. Severity of disease was defined accordingly: 0–149 is remission, 150–219 is low to moderate activity, 220 to 450 is moderate-severe, and over 450 is very severe activity. Patients with CD were selected based on two major criteria: having at most 5 years of disease activity and having a CDAI score under 220 during their last examination, which consists of low to moderate disease activity. Exclusion criteria were patients with active disease flares, CDAI over 220, as well as other known malignancies or auto-immune diseases in both groups as well as complications found via imaging. In addition, patients with undifferentiated colitis, or with a recent history of infectious gastrointestinal diseases were excluded. In addition, matching criteria for gender and age were established. Patients were screened for CRP (C-reactive protein), fecal calprotectin levels, albumin, hemoglobin, leukocytes and platelet counts. CD patients were screened endoscopically, thus only the patients with a SES-CD (Simple Endoscopic Score for Crohn’s Disease) score lower than 6 were included in the study. Furthermore, patients with biological therapies as well as immunomodulators taken in the last 6 months were excluded from the study.

Age- and sex-matched healthy controls were recruited among hospital staff volunteers and individuals undergoing routine health check-ups, provided they fulfilled the same exclusion criteria.

8 patients were excluded due to inadequacies in the preparation protocol, thus, to maintain the matching criteria, the matched samples were excluded as well, therefore 54 patients were included in the study (27 CD vs. 27 healthy patients). Spectra were excluded if they demonstrated severe acquisition artifacts, including markedly distorted baselines, poor signal-to-noise ratio, or non-reproducible spectral fluctuations preventing reliable normalization and alignment. Quality assessment was performed prior to classification and independently of clinical labels. Although 54 matched participants were included clinically, three control spectra were excluded from the final chemometric analysis because they did not meet spectral quality criteria after preprocessing, due to acquisition artifacts. Therefore, the final classification dataset comprised 51 representative spectra: 27 from CD patients and 24 from healthy controls.

### 4.2. Sample Collection and Preparation Protocol

Eight milliliters of blood were collected during hospital admission after overnight fasting, following the same protocol and the same assistant to maintain standards for every blood sample collection. The blood was centrifuged at 4000× *g* RPM for 10 min, separating the serum and storing it into Eppendorf tubes at −80 degrees Celsius, using the same lab technician for each sample. Afterwards, the samples were brought back to room temperature before performing the same analysis.

### 4.3. SERS Substrate Preparation

The SERS substrates consisted of self-assembled plasmonic silver nanoparticles developed within our group [43]. Briefly, the synthesis involved the chemical reduction of a 10 mM aqueous silver nitrate solution with a 30 mM hydroxylamine solution, following the method developed by Leopold and Lendl [44]. A basic pH was ensured by adding a 63.5 mM sodium hydroxide solution. All solutions were prepared using ultrapure water (18.2 MΩ × cm, ELGA Labwater from PURELAB Chorus, High Wycombe, Buckinghamshire, UK). The obtained nanoparticles were subsequently purified and concentrated 10-fold using tangential flow filtration (TFF, Pall Corporation, New York, NY, USA) with a 10 kDa pore size microcapsule.

The fabrication of the solid self-assembled SERS substrates consisted of depositing 1 µL of the concentrated colloid onto a CaF_2_ Raman-grade glass slide (Crystran, Poole, UK), followed by heating at 40 °C for 2 min. After cooling to room temperature, the substrates were used for serum sample measurements.

Detailed physicochemical characterization and analytical validation of these substrates, including morphology analysis through transmission electron microscopy, UV–Vis characterization, hotspot uniformity, enhancement factor evaluation, standard-analyte quality control, and intra-/inter-batch reproducibility assessments, were previously reported by Știufiuc et al. using the same fabrication protocol employed in the present study [45].

### 4.4. RAMAN and SERS Measurements

For SERS measurements 1 µL of serum was deposited onto the surface of the solid substrates and allowed to dry for 30 min at room temperature. The spectra were recorded using a Renishaw inVia Reflex Raman system (Renishaw plc, Wotton-under-Edge, Gloucestershire, UK) composed of a confocal multilaser spectrometer with a spectral resolution of 0.5 cm^−1^, equipped with a 600 lines/mm grating and a charge-coupled device (CCD) camera. Calibration was carried out using an internal silicon reference.

SERS analysis was performed using a 785 nm diode laser and the 50× objective under the following conditions: 5 s exposure time, 2 accumulations, and a laser power of 2 mW at the sample surface. Spectral data processing was conducted using Wire 4.2 software provided by Renishaw (Gloucestershire, UK), and final data analysis was performed using the OriginPro 2019 software platform.

### 4.5. Data Analysis

Spectral data processing and classification were performed using the Python programming environment (v3.8), utilizing the NumPy and Pandas modules for data handling, and Scikit-Learn for the machine learning pipeline. To ensure a uniform feature set across all subjects, the raw spectra were baseline-corrected, resampled to a common 1 cm^−1^ grid using linear interpolation, and normalized using the Standard Normal Variate procedure where each intensity value was centered by the mean and scaled by the standard deviation of that specific spectrum.

For the multivariate analysis, two distinct supervised classification models were employed: Principal Component Analysis-Linear Discriminant Analysis (PCA-LDA) and Partial Least Squares Discriminant Analysis (PLS-DA). For the PCA-LDA pipeline, the number of principal components was selected to explain 99% of the total variance in the training set. The PLS-DA model was configured using 5 latent vectors. The performance of both models was evaluated using a Leave-One-Out Cross-Validation (LOOCV) protocol, necessitated by the sample size (*n* = 51). All dimensionality reduction and model fitting steps were performed within each LOOCV training fold to avoid information leakage. Diagnostic performance was quantified via a confusion matrix to calculate sensitivity, specificity, and accuracy. Additionally, the Area Under the Receiver Operating Characteristic (ROC) Curve (AUC) was calculated to assess the overall diagnostic robustness.

To identify the most discriminatory vibrational bands, Variable Importance in Projection (VIP) scores were extracted from the PLS-DA model. Wavenumbers with a VIP score larger than 1 were considered statistically relevant. These selected bands were further validated using an independent two-sample *t*-test to compare mean intensities between the CD group and the healthy control group. To control the false discovery rate (FDR) arising from multiple comparisons, *p*-values were adjusted using the Benjamini-Hochberg (BH) procedure with a significance threshold set at 5%. Continuous clinical variables were compared between groups using Student’s *t*-test or the Mann–Whitney U test, depending on data distribution. Categorical variables were compared using the chi-square test or Fisher’s exact test, as appropriate.

## 5. Conclusions

Our data suggests that serum-based SERS combined with multivariate analysis may represent a feasible approach for differentiating patients with Crohn’s disease from healthy controls. Several discriminatory spectral bands showed high VIP scores, suggesting that serum SERS captures disease-associated biochemical alterations relevant to Crohn’s disease classification. These findings support further investigation of SERS as a rapid, minimally invasive adjunctive diagnostic tool. Future studies should validate these biomarkers longitudinally in larger, multicenter cohorts and evaluate whether spectral signatures can differentiate Crohn’s disease from ulcerative colitis, infectious colitis, irritable bowel syndrome, and other inflammatory or autoimmune conditions.

## Figures and Tables

**Table 1:** Cohort parameters.

Parameters	Crohn’s Disease Group (*n* = 27)	Healthy Subjects (*n* = 27)	*p* Value
Gender (male)	20 (74.1%)	20 (74.1%)	1
Mean age	31.47 (±5.14)	31.47 (±5.14)	1
Mean hemoglobin count	11.9 (±1.83)	12.8 (±1.98)	0.28
Mean CRP	6.7 (±1.49)	1.8 (±0.45)	0.02
Mean albumin count	3.6 (±0.63)	3.9 (±0.47)	0.43
Mean platelet count	203.4 (±11.4)	197.44 (±18.5)	0.58
Mean leukocyte count	10.8 (±1.45)	8.2 (±1.23)	0.04
Mean Calprotectin Level	83.4 (±18.2)	N/A	N/A
CDAI score (less than 150)	12 (44.4%)	N/A	N/A
CDAI score (150–220)	15 (55.6%)	N/A	N/A
SES-CD (less than 3)	14 (51.8%)	N/A	N/A
SES-CD (3–6)	13 (48.2%)	N/A	N/A

**Table 2:** Multivariate analysis and AUC results with CI 95%.

Metric	PCA-LDA Result (95% CI)	PLS-DA Result (95% CI)
Sensitivity	85.19% (72.14–96.45)	88.89% (75.73–99.18)
Specificity	91.67% (81.82–98.66)	95.83% (83.71–98.23)
Accuracy	88.24% (78.13–97.42)	92.16% (81.93–98.74)
AUC	0.881 (0.742–0.986)	0.937 (0.831–0.997)

**Table 3:** Tentative identification of vibrational bands between CD and healthy patients.

Wavenumber (cm^−1^)	Tentative Molecular Assignment	Tendency (CD vs. HC)	Fold Change	VIP Value	*p*-Value (BH)
498	Uric acid/Cystine	Decreased	0.82	1.54	<0.001
639	Uric Acid/Methionine	Decreased	0.76	1.98	<0.001
728	Adenine/Hypoxanthine	Increased	1.15	1.38	0.006
813	RNA/Phosphodiester	Increased	1.11	1.22	0.012
1008	Phenylalanine (Protein)	Stable	1.02	1.08	0.120 (NS)
1136	Phospholipids/Proteins	Increased	1.31	2.24	<0.001
1205	Tyrosine/Phenylalanine	Decreased	0.88	1.45	0.003
1443	Lipids (CH2 deformation)	Increased	1.22	1.72	0.001
1579	Amide II/Nucleic Acids	Increased	1.38	2.11	<0.001
1657	Amide I (\alpha-helix)	Increased	1.45	2.58	<0.001

## Data Availability

The data supporting the findings of this study are available from the main author and the corresponding author upon reasonable request. The data are not publicly available due to privacy and ethical restrictions related to patient confidentiality.
